# Wetting Behavior of LBE on Corroded Candidate LFR Structural Materials of 316L, T91 and CLAM

**DOI:** 10.3390/ma15010102

**Published:** 2021-12-23

**Authors:** Huiping Zhu, Xiaochao Du, Xudong Liu, Tingxu Yan, Xiaobo Li, Yifeng Wang, Muran Qi, Xu Tu

**Affiliations:** 1 School of Nuclear Science and Engineering, North China Electric Power University, Beijing 102206, China; lxdong947@163.com (X.L.); YTXAn23@163.com (T.Y.); lixiaobo@ncepu.edu.cn (X.L.); wonn333@163.com (Y.W.); qq1938906434@163.com (M.Q.); tuxu13pingan@ncepu.edu.cn (X.T.); 2 College of Science, Three Corges University, Yichang 443002, China; duxc@ctgu.edu.cn

**Keywords:** wetting behavior, lead-bismuth eutectic, corroded steel, corrosion morphology

## Abstract

In this work, the wetting behaviors of lead-bismuth eutectic (LBE) on corroded 316L, T91, and CLAM surfaces were studied. The wettability of LBE on virgin and corroded surfaces were tested at 450 °C by using the sessile-drop (SD) method after immersing the samples in LBE with saturated oxygen concentration for 400, 800, and 1200 h at 450 °C. Additionally, the morphology, as well as element distribution of the corrosion structure, were characterized by scanning electron microscope (SEM) and energy-dispersive X-ray spectroscopy (EDS). The results showed that the virgin samples of three materials are non-wetting to LBE, and the formation of corrosion structures further reduces the wettability. Besides, the thickness of the corrosion layer formed on the 316L surface grew more slowly than the other two steel, which results in better corrosion resistance of austenitic steel 316L than that of ferritic/martensitic steels T91 and CLAM at 450 °C. Meanwhile, the morphology and distribution of corrosion products are important factors affecting the wettability of the steel surface. The formation of corrosion products with high roughness as well as disorder results in a significant reduction in surface wettability.

## 1. Introduction

Lead-bismuth eutectic (LBE) has been proposed as a coolant material for a lead-cooled fast reactor (LFR) and an accelerate driven system (ADS) [1,2]. In practical application, structural materials are in direct contact with LBE; the wettability of LBE on the surface of structural materials has an important impact on the performance of materials. At first, the wettability between material surface and LBE is a key parameter affecting the liquid metal embrittlement (LME) behavior of the structural materials. Generally, materials with poor LBE wettability possess a lower probability of LME [3,4,5,6,7,8]. Secondly, the surface wettability is directly related to the corrosion properties of the materials. Poor wettability of the surface hinders the diffusion and reaction of elements between the corrosive medium and the material matrix, which is beneficial to improve the corrosion resistance of the material [9,10,11,12]. In addition, the surface wettability of structural materials is an important factor affecting the fluid pressure drop and wall heat transfer, which will decrease with the deterioration of wettability [13,14,15,16,17]. Therefore, the wettability of structural materials to LBE plays a significant role in the performance evaluation of structural materials.

By now, a lot of data have been accumulated concerning the wettability between liquid metals and materials. However, research on the wettability between LBE and stainless-steel structural materials in LFR and ADS are still very limited. The existing studies mainly focus on the differences between the wettability of lead and LBE on structural materials [18,19,20], the main factors and mechanisms affecting the wettability [18,20,21,22], and the influence of surface wettability on LME of structural materials [3,4,5,6,7,23]. Among these studies, D. Giuranno et al. investigated the wetting behavior of the LBE and pure Pb on AISI 316L substrate over the temperature range from 623 K to 773 K [18]. They found that the contact angle of LBE on the sample was smaller than that of pure Pb, and the contact angle decreased with increasing temperature. Besides, J. Liu [19] found that the contact angle of molten LBE on T91 steel was larger than that of Pb at the same temperature, and there was no obvious reactivity on the interface of both systems. P. Protsenko [20] illustrated that the similar contact angle measured on the pure iron surface for Pb and LBE are induced by the resemblance of interactions of Pb and LBE. Meanwhile, Giuranno [18], P. Protsenko [20], Z. Qi [21], and Z.Y. Wu [22] pointed that surface roughness, metallurgical state of the substrate material, ambient temperature, and atmosphere are the most important factors affecting the wettability of LBE on materials. Besides, it was clarified [4,23] that the intimate contact between LM and the material surface is the necessary condition that has to be reached to observe LME. The formation of an oxide layer on the material surface during the corrosion process plays an important role in both the wettability and LME behavior [4]. Overall, the existing research results confirm the following important contents:

1. Typical stainless steel such as 316L and T91 are neither wetting to LBE nor Pb. The differences are mainly induced by the different surface tension of LBE and Pb droplets or the reactivity of liquid metal (LM) on various steel at a certain temperature.
2. The high temperature will increase the wettability of stainless steel to LBE. High roughness will make the wetting surfaces more wet and non-wetting surfaces less wet.
3. The decrease in surface wettability of LBE on structural materials reduces the elements’ interdiffusion and reaction, which is beneficial to inhibit the occurrence of LME during service.

It is important to note that these findings are based on the discussion of the wettability of virgin steel samples to LBE. In practical applications, due to LBE corrosion, an oxidation structure will form on the surface of the steel. The oxide corrosion structure separates the LBE from the material matrix and directly contact LBE. Therefore, revealing the influence of the oxide structure formed during the corrosion process on the wettability of LBE is of great significance for clarifying the embrittlement of the material in the service process. In this paper, the wettability of LBE on the surface of three candidates steel after corrosion is studied, and the reasons leading to the differences in wettability of materials are analyzed.

## 2. Materials and Methods

### 2.1. Materials

Three typical candidate structural materials 316L, T91, and CLAM of LFR were selected. The CLAM steel is provided by the Institute of Nuclear Safety Technology, Chinese Academy of Sciences (CAS), Hefei, China. The nominal composition of commercial 316L and T91, as well as CLAM, is shown in Table 1. Before corrosion, the three materials were cut into small pieces of 15 × 6 × 1.5 mm^3^ in size. Surfaces of the samples were mechanically polished, initially with sandpapers of varying grits, and finally to a fine mirror finish with a 0.5 μm diamond spray. Subsequently, the surfaces were ultrasonically cleaned in alcohol and acetone in order. All the samples were vacuum stored before corrosion.

### 2.2. Corrosion Experiments

**Figure 1:**
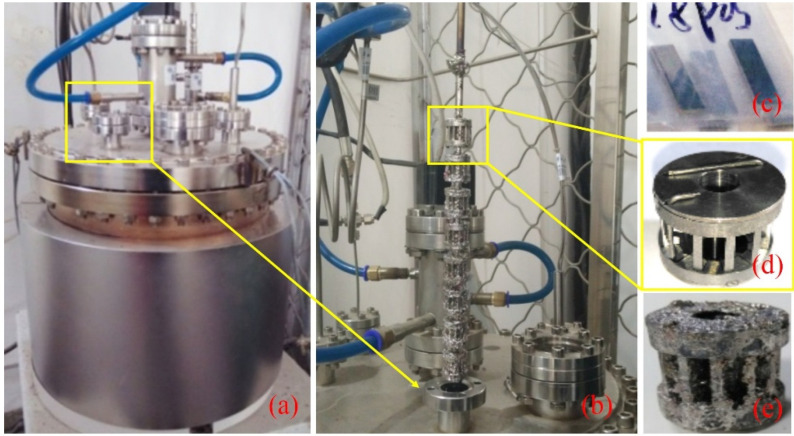
Pot-type multifunctional LBE corrosion platform: (**a**) LBE corrosion tank, (**b**) sample holder, (**c**) original samples, (**d**) corrosion unit, (**e**) corroded corrosion unit [24].

The corrosion experiment was performed on the pot-type multifunctional LBE corrosion platform of North China Electric Power University, Beijing, China, as shown in Figure 1. The corrosion samples were mounted on the sample holder with 8 sample units. During the corrosion process, all the 8 units were immersed in static LBE at 450 °C. The oxygen concentration of LBE was controlled at a saturated value of about 3.14 × 10^−4^ wt.% [18]. Two corrosion sample units were taken out every 400 h, and the last two units were spares. The longest corrosion time was 1200 h.

To observe the surface morphology of the material after corrosion, the surface of the material was cleaned in hot glycerin. Most of the residual LBE was washed away during this process. Then, the sample was immersed in the mixed solution of acetic acid, hydrogen peroxide, and ethanol with the volume ratio of 1:1:1 for 12 h to remove the remaining LBE on the surface of the material. After that, the surface morphology was collected directly by using a scanning electron microscope (SEM). The sample used for cross-section observation was firstly cut into pieces and then embedded with epoxy resin. After polishing, the cross-sectional morphology and elements distribution were obtained by SEM and energy-dispersive X-ray spectroscopy (EDS). Besides, the roughness of corroded samples was measured by using a mini smart surface roughness measuring instrument.

### 2.3. Wetting Test

**Figure 2:**
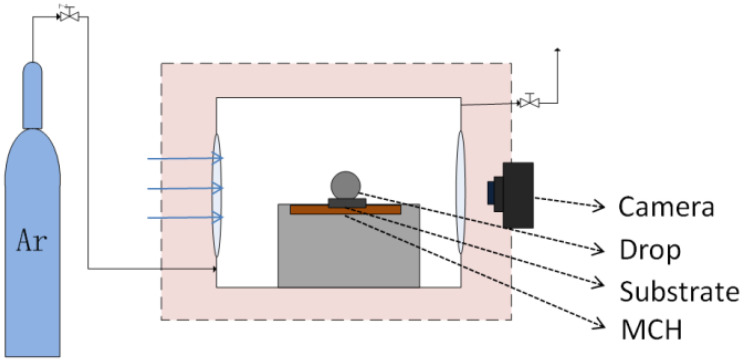
The schematic of the contact angle measuring device [24].

The wetting test was carried out in a smart contact angle measuring device by using the sessile drop method. The schematic diagram is shown in Figure 2.

The droplet sample was 0.25 g with an error of ±0.01 g. Before experiments, LBE droplets were pre-melted in heated glycerol at about 200 °C and then cooled in cold glycerol. The remaining glycerol on the sample surface was cleaned by soaking in acetone for 3–5 min. Then, the LBE droplet was sealed and stored.

When the test began, one sample was placed on the horizontal metal plate, which was a metal ceramics heater (MCH), and the LBE droplet was placed on the center position of the sample surface. Argon atmosphere was maintained in the chamber during the test. After the temperature raised to 450 °C, the status was maintained for 20~30 min. During the process, the droplet could melt and form a drop with equilibrated geometry. Then, the image of the sessile droplet was taken by the camera.

**Figure 3:**
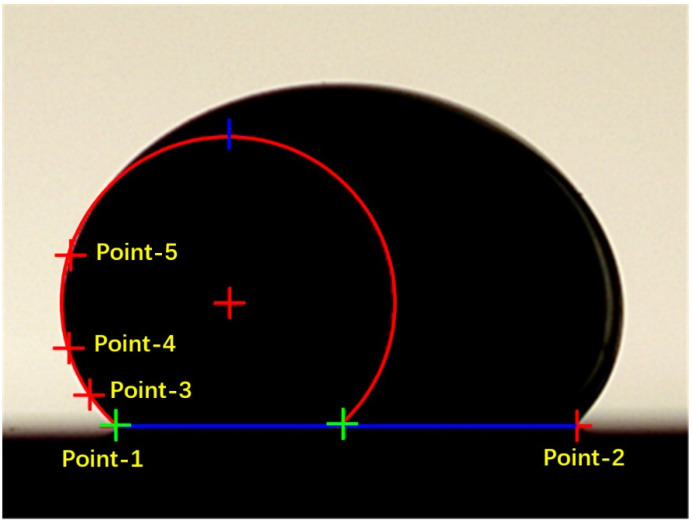
Image processing: an example of five-point fits [24].

Graphical analysis software JC2000 (G1, Shanghai Zhongchen Digital Technic Apparatus Co., Ltd, Shanghai, China) was used to analyze the droplet photos. Contact angles were automatically determined by a method of five-point fit, as shown in Figure 3. Taking into account the operation error, more than 10 images of contact angle for each group of corrosion samples were collected. The average value of the 10 images was determined as the contact angle of the sample.

## 3. Results

### 3.1. Contact Angle

**Figure 4:**
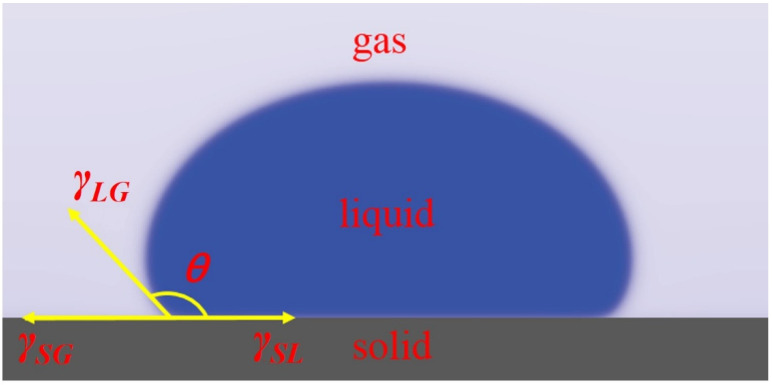
The contact angle of a liquid drop on the solid surface [26].

The contact angle is the conventional standard for measuring surface wettability [25]. The contact angle refers to the angle *θ_c_* between the tangent of the gas–liquid interface at the intersection of the gas, liquid, and solid that passes through the liquid and solid–liquid boundary line. It is a measure of the degree of wetting, as shown in Figure 4. The wetting process is related to the interfacial tension of the system. When the droplet reaches equilibrium on a horizontal solid surface, the contact angle and the interfacial tension follow Young’s Equation [26]:

(1) $$\cos\;\theta_{c}=\frac{\gamma_{SG}-\gamma_{SL}}{\gamma_{LG}}$$

Among them, *γ_SG_*, *γ_SL_*, and *γ_LG_* represent the free energy of solid–gas, solid–liquid, and liquid–solid interfaces, respectively. When *θ_c_* = 0°, it is defined that the liquid is completely wetted on the solid surface. When 0 < *θ_c_ ≤* 90°, the solid surface is defined as wetting surface; when 90° ≤ *θ_c_* ≤ 150°, the solid surface is defined as a non-wetting surface. When *θ_c_* ≥ 150°, the solid surface is defined as a super non-wetting surface.

**Figure 5:**
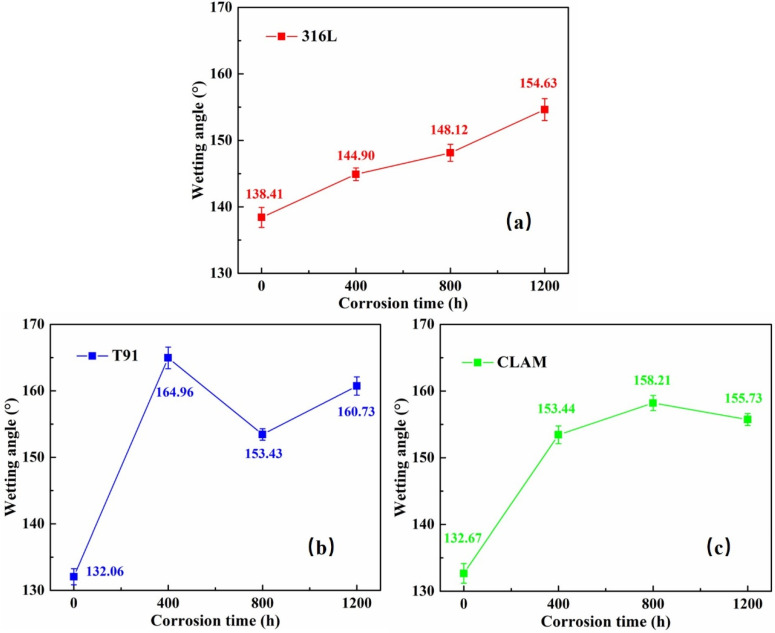
The statistical wetting angles of samples before and after corrosion at 450 °C: (**a**) 316L, (**b**) T91, (**c**) CLAM.

The statistical contact angles of the LBE droplet on the sample surfaces before and after corrosion are shown in Figure 5.

Before corrosion, the contact angle of LBE on the surfaces of the three materials is greater than 130°, indicating that the original materials are non-wetting to LBE. Compared with two ferrite/martensite (F/M) steel of T91and CLAM, the austenite steel of 316L shows lower wettability to LBE at 450 °C. After the corrosion, with the formation of the corrosion structure, the wettability of the surfaces to LBE changed significantly. The contact angles of LBE droplets on the surface of all corroded samples are larger than that of the virgin samples, which means the formation of corrosion structure leads to the degeneration of surface wettability. For the 316L corrosion sample, as the corrosion time increases, the contact angle gradually increases with the corrosion time. For the 316L sample corroded for 1200 h, the contact angle is greater than 150°, which reaches the super non-wetting range. For T91 and CLAM steel, the surface contact angles of all corrosion samples are greater than 150°. As the corrosion time increases, the contact angle fluctuates around 155°.

### 3.2. Cross-Section of the Corrosion Layer

**Figure 6:**
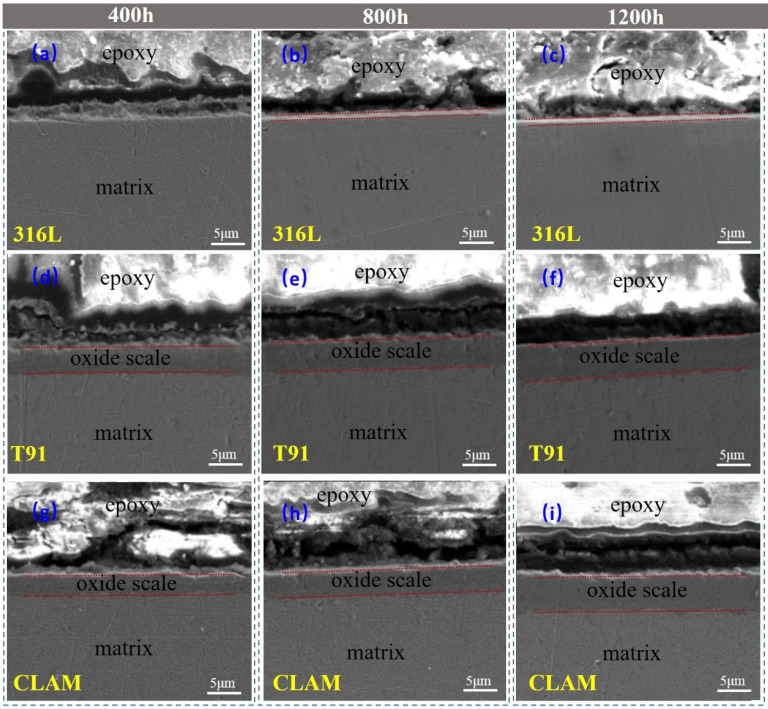
Cross-section morphology of the corroded samples: (**a**) 316L-400h, (**b**) 316L-800h, (**c**) 316L-1200h, (**d**) T91-400h, (**e**) T91-800h, (**f**) T91-1200h, (**g**) CLAM-400h, (**h**) CLAM-800h, (**i**) CLAM-1200h.

The cross-sectional morphology of corroded samples is shown in Figure 6. It shows that in the LBE with saturated oxygen concentration, the corrosion of the three materials occurred in different degrees.

Compared with F/M steel T91 and CLAM, austenitic steel 316L shows better corrosion resistance. For the 316L sample corroded for 400 h, no obvious corrosion structure was found on the surface of the material. As the corrosion time increased, the corrosion layer on the surface of the material grew slowly. After 1200 h of corrosion, the thickness of the surface corrosion layer was still less than 1 μm.

For the corroded samples of T91 and CLAM, obvious corrosion structures were formed on the surfaces. For T91, after 400 h, the thickness of the corrosion layer on the surface of the material was about 3.95 (±0.15) μm. As the corrosion time increased, the corrosion layer gradually thickened. After 1200 h, the thickness of the corrosion layer reached 5.28 (±0.23) μm. The growth rate of the corrosion layer was about 9.88 × 10^−3^ μm/h before 400 h. After that, the growth rate of the corrosion layer was significantly reduced, which was about 1.66 × 10^−3^ μm/h during 400–1200 h. This shows that the formation of the corrosion layer significantly inhibits the corrosion of the material surface. For CLAM, a corrosion layer of 3.26 (±0.18) μm was formed on the surface of the material after 400 h, and the corrosion layer grew to 5.39 (±0.19) μm after 1200 h. The formation of the corrosion layer also inhibits the corrosion of the material surface. The growth rate of the corrosion layer reduces from 8.15 μm/h before 400 h to 2.66 μm/h in 400–1200 h.

**Figure 7:**
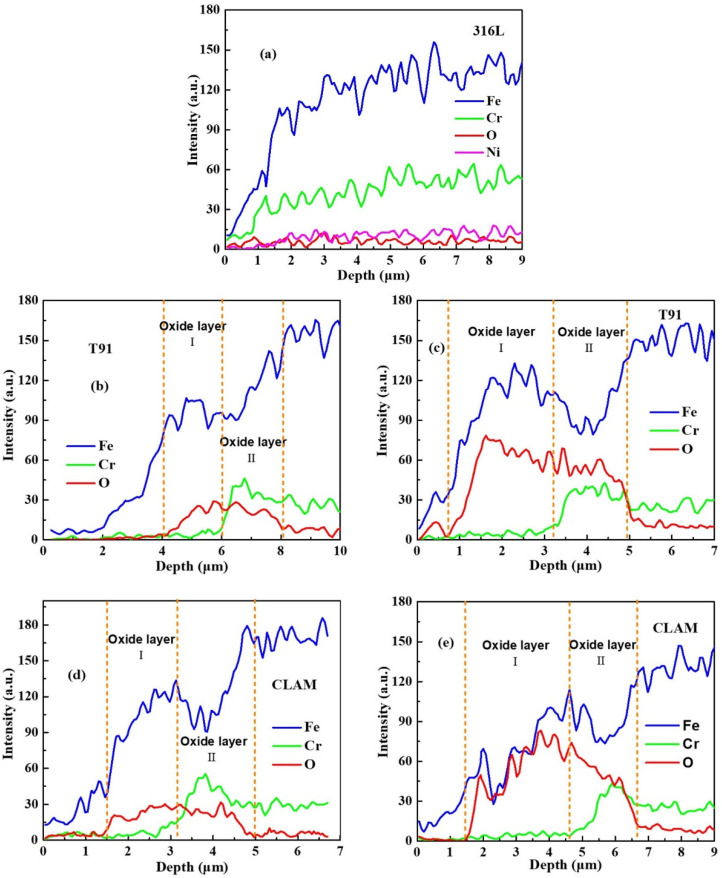
The elements distribution of cross-section of (**a**) 316L-1200 h, (**b**) T91-400 h, (**c**) T91-1200 h, (**d**) CLAM-400 h, and (**e**) CLAM-1200 h.

Figure 7 shows the depth-dependent distribution of the main matrix elements in the corroded samples. For the 316L samples, the result is identical to the typical distribution of elements in the virgin samples’ cross-section, from which it is hard to distinguish whether there is a corrosion structure on the surface. For the T91 and CLAM samples corroded for 400 h, the corrosion layer possesses an obvious double-layer structure. The outer layer (Oxide layer I) is mainly Fe_3_O_4_ while the inner layer (Oxide layer II) is (Fe, Cr)_3_O_4_ spinel structure. With the increase in corrosion time, the oxide layer on the surface of the material gradually thickens, and the inner and outer oxide layers gradually grow. Compared with the existing literature data [27,28,29], it can be found that although the composition of the corrosion structure of T91 and CLAM is consistent with the prior data, its thickness is significantly smaller. Considering that the corrosion tank in the experiment is a sealed structure, the dissolved oxygen in LBE can be consumed by the tank material, which will induce the reduction of the oxygen concentration in LBE and result in a lower oxygen content corrosion experiment. This may be the reason for the formation of a thinner oxide layer in this work.

### 3.3. Surface of Corroded Samples

**Figure 8:**
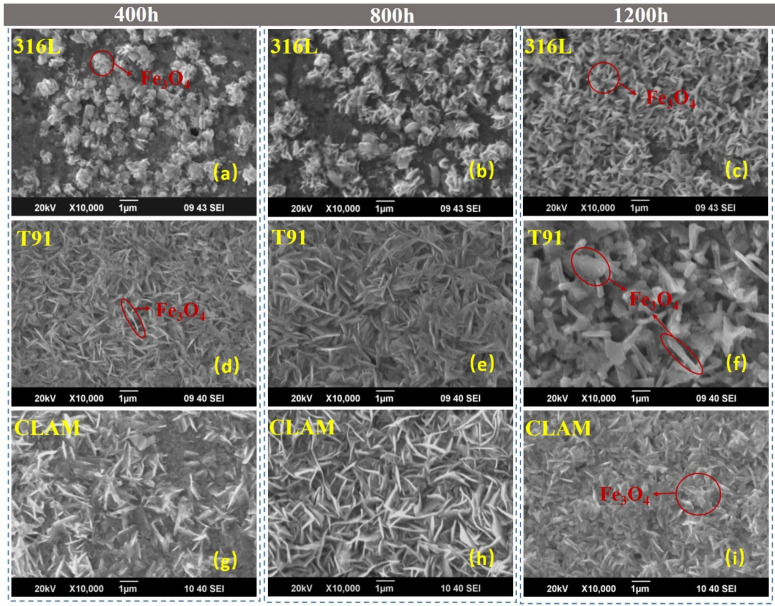
Surface morphology of the corroded samples: (**a**) 316L-400h, (**b**) 316L-800h, (**c**) 316L-1200h, (**d**) T91-400h, (**e**) T91-800h, (**f**) T91-1200h, (**g**) CLAM-400h, (**h**) CLAM-800h, (**i**) CLAM-1200h.

As shown in Figure 8, the surface morphology of the three materials changed significantly after corrosion.

For the 316L corrosion sample, although no obvious corrosion layer was found in the cross-sectional observation, it can be seen that the surface of 316L was partially oxidized after 400 h, and a cluster-like surface corrosion structure was formed locally. The cluster-like structure was composed of fine needle-like oxides. After 400 h, the smallest clusters on the surface of the samples were only 0.22 μm, the largest clusters were about 1.64 μm, and the average size was about 0.69 μm. With the increase in corrosion time, the size of the cluster-like structure increased significantly. For the sample corroded for 800 h, the largest cluster size was about 2.07 μm, while the average cluster size reached 1.13 μm. At this time, there were still parts of the surface that were not covered by the corroded structure. When the corrosion time reached 1200 h, the cluster-like corrosion structure continued to grow and join together. The surface of the material was covered by needle-like corrosion products with a coverage rate of about 95%. The maximum length of the needle-like structure was about 1.20 μm, the average size was about 0.58 μm. The elemental composition of the needle-like structure was tested by EDS, which showed that was mainly Fe_3_O_4_.

After 400 h of corrosion, the T91 surface has been covered by needle-like corrosion products. The EDS scan results showed that the corrosion products are Fe_3_O_4_. The average length of needle-like corrosion products was about 0.71 μm. As the corrosion time increased, the needle-like corrosion product grew longer, and its density decreased. When the corrosion time reached 1200 h, needle-like corrosion products grew into rods, and flake-like corrosion products appeared. The average size of the corrosion product increased to 0.91 μm, and its density decreased from the value of 4.292 × 10^8^ cm^−2^ at 400 h to 1.295 × 10^8^ cm^−2^ at 1200 h, which was about 2 times less.

Slightly different from T91, the surface of CLAM corroded for 400 h is mainly composed of needle-like and flake-like corrosion products. The average size of the corrosion products was about 0.57 μm, and the longest needle-like corrosion product was about 1.79 μm. When the corrosion reached 800 h, needle-like corrosion products with the same morphology as T91 corroded for 800 h were formed on the surface of CLAM. The flaky corrosion products generated on the sample corroded for 400 h were no longer visible, only the needle-like products with an average length of about 0.87 μm existed on the CLAM surface corroded for 800 h. It is worth noting that with the increase in corrosion time, the corrosion products generated in the previous period clearly adhered; they gathered together and gradually formed new massive and flaky corrosion products at 1200 h. Only part of the original needle-like corrosion products remained on the surface, and its length was significantly reduced by the effect of adhesion. At this time, the longest needle-like corrosion product was about 1.25 μm, and the average size of the overall corrosion product was smaller, about 0.52 μm. EDS measurement shows that although the morphology of the corrosion structure on the surface of the material had changed, it was still Fe_3_O_4_.

## 4. Discussion

### 4.1. Factors Affecting Wettability

**Figure 9:**
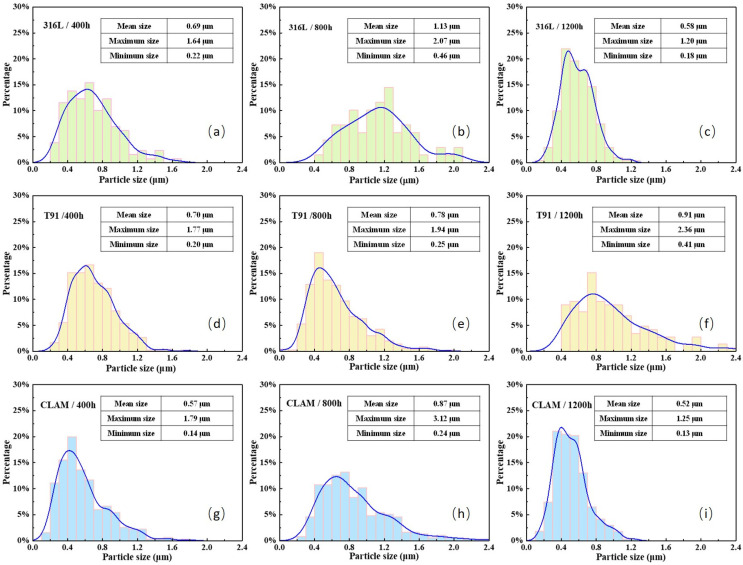
Statistical size distribution of corrosion products on the corroded sample surface: (**a**) 316L-400h, (**b**) 316L-800h, (**c**) 316L-1200h, (**d**) T91-400h, (**e**) T91-800h, (**f**) T91-1200h, (**g**) CLAM-400h, (**h**) CLAM-800h, (**i**) CLAM-1200h.

To clarify the main factors that affect the wettability of samples, statistical information of the size distribution, density, structure of the corrosion products, surface roughness, and oxide layer thickness was obtained using the software Nano-Measure, and the results are shown in Figure 9 and Table 2.

Among the factors that affect surface wettability, the microstructure, element composition, and surface characteristics of the material are the internal factors that affect the surface wettability to liquid metal. For the three materials studied in this article, the microstructure is different. For 316L austenitic steel, it is fcc structure, the F/M steel T91 and CLAM are bcc structures. In addition, the element composition of austenitic steel 316L is different from that of F/M steel T91 and CLAM. Under the same surface polishing conditions, there were significant differences in their surface wettability to LBE, especially between austenitic steel and F/M steel. It shows that the LBE wettability of the 316L surface is lower than the two F/M steel. The high content in Cr, Ni, Mo, and the fcc structure with higher lattice density may be beneficial to reduce the wetting of LBE on the material surface.

After corrosion, the oxidized structure formed on the surface of the material isolates the LBE from the material matrix. It can be seen that when the surface corrosion structure completely covers the material surface, the substance directly facing the LBE becomes an oxide layer of Fe_3_O_4_. The formation of the oxide layer increases the roughness of the material surface, which leads to the deterioration of wettability. In addition, the size, density, and structure of the corrosion products also affects the wettability of the surface. Corrosion products of high density, small size, and small complex structures can significantly increase the degree of disorder on the surface of the material by improving the density and reducing the size of the interstices on the material surface. The increase in surface roughness and disorder enhances the free energy of the material surface. According to Wenzel [30], to reduce the energy of the LBE-steel system, the contact angle between the metal droplet and the surface of the stainless steel will increase, inducing the material surface to reduce the wettability of LBE. It is worth noting that the changing trend of any of the aforementioned parameters is not completely consistent with that of the wetting angle between LBE and material surface. Therefore, the wettability of the material surface is a result of the combined effects of these factors. To establish a more accurate quantitative model between the parameters and wettability, more wettability results and surface structure analysis results of corrosion samples are also needed.

### 4.2. Wettability and Corrosion

The corrosion of structural steel is closely related to the dissolved oxygen content in LBE, the flow rate of the liquid metal, the temperature, and the characteristics of the material surface [31]. In LBE with low oxygen concentration, dissolution corrosion is the main corrosion mechanism. This effect, on one hand, dissolves the material matrix, on the other hand, within a certain temperature range, it will cause lead-bismuth atoms to infiltrate the alloy along dislocations or grain boundaries and sub-grain boundaries, resulting in a sharp drop in the plasticity of the material [27,32,33]. In LBE with a high oxygen concentration, oxidation corrosion plays a dominant role. This effect leads to the formation of a complex oxide layer structure on the surface of the material. For long-term corrosion, the excessively thick oxide layer structure will cause a decrease in thermal conductivity and an increase in the temperature inside the cladding [30]. In addition, the flow-accelerated corrosion effect induced by the flow field will accelerate the corrosion process and induce more complex forms of corrosion [34,35].

In this paper, the surface of the material undergoes different degrees of oxidative corrosion with the increase in corrosion time. Compared with F/M steel T91 and CLAM, 316L, which has lower wettability to LBE, has a lower growth rate of the surface corrosion layer, which is determined by characteristics such as the original structure and composition of the material. When the oxide layer completely covers the surface of the material (corrosion for 1200 h), the surface free energy between the magnetite layer with loose porous structure and LBE is lower, leading to a decrease in the wettability between the two. In addition, the formation of the surface corrosion layer separates the LBE from the material matrix, and its non-wetting characteristic is not conducive to the interdiffusion of elements between LBE and the matrix of material, which is beneficial to reduce the corrosion rate of the material surface. However, this hindering effect is not permanent [8]. In practical applications, the flowing LM helps LBE to overcome the energy barrier of the oxide structure on the uneven surface, leading to the penetration of the LBE. Therefore, the real protection of the material also depends on the formation of the dense and stable spinel oxidation structure of the inner layer [28,35,36].

### 4.3. Wettability and LME

In practical applications, the LME effect occurs at the wetted crack nucleation on the solid metal surface. The necessary condition for LME to occur required an intimate contact between the liquid metal and the solid metal, and the application of plastic deformation [5,8]. Therefore, the wettability between liquid metal and structural material can be used as an important reference for predicting the LME sensitivity of materials [37]. In this article, the original surfaces of the three materials are all non-wetting to LBE, indicating that the adsorption between the surface of the material and LBE is weak. Hence, the material can be considered to have a low LME tendency in the absence of other influencing factors (such as the environment temperature, the structure characteristics and mechanical properties of the material, the strain rate that the material suffered, etc.). In addition, when a corrosion structure is formed on the surface of the material, the wettability of the material surface to LBE is further reduced. On the other hand, the isolation effect of the corrosion layer on the LBE and the material matrix also avoids direct contact between the two, thereby inhibiting the occurrence of LME [3,4,6,7,18]. When the stress load on the surface is not enough to destroy the integrity of the oxide layer, or the speed of the oxide layer destruction at a slow strain rate is lower than its self-healing speed, the surface oxide layer will provide continuous protection to the material matrix [6]. However, when the oxide layer is destroyed and LBE penetrates to the structural steel matrix, LME occurred at crack tips, causing fast crack propagation.

## 5. Conclusions

The wettability of 316L, T91, and CLAM steel to LBE were studied before and after corrosion in the LBE with saturated oxygen concentration at 450 °C. By using the sessile-drop method, the contact angle between the LBE droplet and sample surface was tested at 450 °C. Combined with the analysis results of SEM, the interaction between surface wettability and corrosion structure growth was discussed in this paper. The conclusion can be drawn as follows:

1. In addition to surface roughness, the increase in surface disorder caused by the growth of corrosion products is also an important factor leading to the decrease in wettability of the materials to LBE.
2. The oxidation structure formed on the surface of the corroded material reduces the wettability of the material surface to LBE and prevents direct contact between the material matrix and LBE, which is beneficial to decrease the corrosion rate and LME tendency of the material.
3. The wettability of the material surface can be used as an important basis for predicting the LME tendency of different materials under certain corrosive environments.

## Figures and Tables

**Table 1:** Main chemical composition of investigated steel (in wt.%).

Materials	Fe	Cr	C	Si	Ni	V	Mn	N	P	S	Mo	Nb	W	Ta	Al	Ti
316L	Bal.	16	0.022	0.51	10.1	-	1.58	-	0.029	0.016	2.2	-	-	-	-	-
T91	Bal.	8.2	0.09	0.31	0.06	0.2	0.39	0.44	0.1	0.02	0.93	0.07	-		0.05	0.02
CLAM	Bal.	8.91	0.12	0.066	0.043	0.2	0.35	0.0084	0.003	0.002	-	-	1.47	0.14	0.027	-

**Table 2:** Statistical density, structure, roughness, and oxide layer thickness of the corroded sample surface.

Materials	Corrosion Time (h)	Corrosion Product Structure	Density (cm^−2^)	Roughness (μm)	Oxide Layer Thickness (μm)
316L	0	-	-	0.024 (±0.008)	-
	400	Clusters	1.166 (±0.157) × 10^8^	0.451 (±0.251)	<1
	800	Clusters	6.261 (±0.112) × 10^7^	0.635 (±0.347)	<1
	1200	Needles	4.117 (±0.214) × 10^8^	0.485 (±0.297)	<1
T91	0	-	-	0.018 (±0.004)	-
	400	Needles	4.292 (±0.397) × 10^8^	2.583 (±0.854)	3.95 (±0.15)
	800	Needles	4.166 (±0.318) × 10^8^	2.712 (±0.426)	4.65 (±0.20)
	1200	Rods/Flakes	1.295 (±0.145) × 10^8^	2.657 (±0.757)	5.28 (±0.23)
CLAM	0	-	-	0.026 (±0.006)	-
	400	Needles/Flakes	2.644 (±0.263) × 10^8^	2.251 (±0.815)	3.26 (±0.18)
	800	Needles	3.183 (±0.309) × 10^8^	2.483 (±0.765)	4.06 (±0.27)
	1200	Needles/blocks	3.064 (±0.246) × 10^8^	3.095 (±0. 631)	5.39 (±0.19)

## Data Availability

All data is contained within the article.
